# Bridging Barriers: Engaging Ethnic Minorities in Cardiovascular Research

**DOI:** 10.3390/healthcare13111217

**Published:** 2025-05-22

**Authors:** Nora Bacour, Simran Grewal, M. Corrette Ploem, Jeanine Suurmond, Robert J. M. Klautz, Nimrat Grewal

**Affiliations:** 1 Department of Cardiothoracic Surgery, Amsterdam University Medical Center, Location AMC, 1105 AZ Amsterdam, The Netherlands; 2 Department of Orthopaedic Surgery, Onze Lieve Vrouwe Gasthuis, 1091 AC Amsterdam, The Netherlands; 3 Department of Ethics, Law & Humanities, Amsterdam UMC, University of Amsterdam, 1012 WP Amsterdam, The Netherlands; m.c.ploem@amsterdamumc.nl; 4 Department of Primary and Community Care, Radboudumc, 6525 GA Nijmegen, The Netherlands; 5 Department of Cardiothoracic Surgery, Leiden University Medical Center, 2333 ZG Leiden, The Netherlands

**Keywords:** ethnic minorities, cardiovascular research, health equity

## Abstract

**Background/Objectives**: We address the ongoing under-representation of ethnic minority groups in cardiovascular research in this opinion paper—a challenge that limits both scientific validity and equitable healthcare outcomes. We aim to outline the underlying causes of this issue and propose concrete strategies to address it. **Methods**: To engage ethnic minorities in cardiovascular research, we thoroughly studied the existing literature and gathered expert opinions to identify barriers and formulate practical solutions. **Results**: Our findings highlight the need for a multifaceted approach, including culturally appropriate educational outreach, interactive multimedia information, community ambassador programs, and improved, but ethically sound, ethnicity registration practices. **Conclusions**: To promote ethnic minority participation in cardiovascular research, a thorough improvement plan is required. Our proposed solutions, which align with insights from the current literature, suggest that addressing cultural, structural, and informational barriers can help achieve a more representative and inclusive participant population. This is an essential step towards improving cardiovascular outcomes for all.

## 1. Introduction

Ethnic diversity is now a common aspect of European societies, including in the Netherlands. According to a recent report from the Dutch Central Statistical Office 12% of the Dutch population have at least one non-native parent, while around 16% of the population is foreign-born themselves [1]. Nevertheless, ethnic minorities are frequently under-represented in health research globally, which results in a lost opportunity to address health disparities and develop personalized interventions [2,3,4,5]. However, for good and equitable healthcare—gradually evolving into more personalized therapies and interventions—it is essential that it is based on research in which all relevant ethnic minority groups are represented [3]. Risk stratification for medical diseases and their treatment strategies must take into account a number of individual characteristics, including clinical, lifestyle, and genetic factors [6,7,8]. Research plays a pivotal role in this process by uncovering potential risks factors of cardiovascular diseases. While extensive research has been performed on sex differences in cardiovascular medicine [9], ethnic disparities remain largely unknown. Despite the limited research available, existing studies have highlighted significant racial and ethnic disparities in cardiovascular health presentation and outcomes, including differences in prevalence, post-operative complications, and mortality rates of various diseases [10,11,12,13,14]. Taken together, this raises valuable questions about the role of ethnic background in cardiovascular medicine and underscores the need for comprehensive research in this area.

When referring to racial or ethnic disparities, it is important to consider differences between populations that arise from biological predispositions, where certain groups are genetically more at risk of developing (severe) cardiovascular diseases than other groups [15,16], but also to consider differences between populations that arise from traditions, migration history, lifestyle, diet, and values, where an ethnic minority status may also influence access to healthcare, health literacy, socioeconomic factors, and health outcomes [16,17]. Nonetheless, as both race and ethnicity are social constructs, they should be approached with careful consideration and sensitivity.

Investigating the underlying causes and possible solutions for ethnic minority under-representation in clinical research could contribute to the realization of improved treatment strategies for individual patients. In this way, all patients in need of cardiovascular care can expect appropriate care, i.e., regardless their ethnic background.

## 2. Problem Statement

Research has identified disparities in health outcomes among ethnic groups. It is essential to deepen the current understanding of the factors contributing to these disparities to ensure that all our patients receive optimal care, tailored to their needs. The main problem, however, is that ethnic minorities are underrepresented in health research for a number of reasons. According to some studies, language barriers, cultural differences, and mistrust in the healthcare system are among the issues minority group members often face, which may ultimately lead to lower participation rates [18]. In contrast, one study found that ethnic minorities’ non-participation in healthcare is often due to a lack of access, rather than distrust [5]. Furthermore, experiences of discrimination among ethnic minorities may contribute to underutilization of healthcare services [19].

These findings fuel the broader problem of decreased participation in medical research as a result of digital illiteracy and obstacles such as a lack of accessible and culturally sensitive healthcare resources. At the same time, research suggests that physicians who are not involved in caring for ethnic minority patients have (un)conscious biases that influence their perceptions of these patients [20], which also leads to fewer participants from these groups being included in research [21]. Together, these factors may complicate the ability to create representative, large-scale study populations, which is essential for advancing research on ethnic minorities.

## 3. Possible Solution Directions

Promoting ethnic minority participation in clinical research requires the application of multiple strategies to overcome these obstacles (Table A1).

### 3.1. Educational Outreach

First of all, it is essential to conduct an educational outreach to support minority groups. Many patients of minority ethnic descent may be unaware of the value of clinical research or feel intimidated by complex medical terminology and lengthy consent forms [18]. Therefore, we suggest to use simplified, culturally, and linguistically appropriate communication, all while trying to raise awareness about the importance of clinical as well as observational research, and the importance of the willingness of ethnic minority groups to participate. Additionally, a trust-building approach is essential improve participation rates among ethnic minority groups [22]. This can involve providing feedback on the outcomes of research, translating research materials into multiple languages and working with staff members who are culturally competent and aware of the unique needs and concerns these groups may have regarding cardiovascular care. For example, multilingual doctors could explain the importance of clinical research in a language that feels familiar and comfortable for patients. Possibly, conversational AI as another a tool to overcome language barriers, could also play a substantial role here [23,24]. However, it is crucial to recognize the ethical considerations and potential limitations of using such AI-tools. Engaging with ethnic minorities in the development of these AI models becomes imperative, as we want to avoid the risk of these models being influenced by health inequalities and to ensure equitable benefits [25].

Furthermore, educating healthcare professionals on the prevalence of various cardiovascular diseases among ethnic groups is crucial [26]. Healthcare professionals should also strive to gain a more profound understanding of the cultural beliefs surrounding certain diseases, treatment options, and medicines. Dietitians could make a valuable contribution by providing nutritional guidance tailored to the specific food preferences of various ethnic communities. In turn, this could help foster more trust in healthcare professionals, ultimately increasing the likelihood that ethnic minorities will participate in scientific research.

### 3.2. Interactive Multimedia Information

Secondly, incorporating interactive multimedia information into the study process can help solve issues of digital illiteracy and language barriers, as well as increase participation rates and foster trust [27]. Brief videos, podcasts, and infographics may be used to simplify medical research. To meet the needs of individuals with varying linguistic demands, information would need to be offered in multiple languages. Again, AI could play an important role here, as an AI-powered digital platform may be beneficial to personalize outreach by clarifying medical terms while adapting messages to cultural norms [28]. Furthermore, this platform may provide dynamic, interactive consent forms with video explanations.

Previous research has shown that implementing mobile health apps could further enhance research participation by advising patients with health-related questions, giving real-time updates on research progress, and reminding patients of community health events [29]. Developing such applications to be culturally attuned and tailored to address the specific needs of each ethnic group—in collaboration with the target audience—not only empowers participants to track their health data but also provides researchers with valuable insights to advance further research, in line with previous recommendations on electronic health interventions for underserved communities [30]. Moreover, including gamification elements, such as badges for participation milestones, might encouraged long-term participant engagement and data collection.

Concurrently, researchers can continuously enhance their study approaches by creating a feedback system where participants can share their experiences and recommend improvements. This suggestion could be put into practice by including anonymous surveys, ratings, or direct communication with research staff. As a result, researchers may be able to adapt to the needs of minority communities.

### 3.3. Community Ambassadors’ Program

Following, a community ambassadors’ program can play a critical role in engaging minority populations. This effort could help to appropriately address cultural sensitivities, while at the same time promoting trust by recruiting respected leaders from ethnic communities to act as intermediaries between researchers and participants. Besides promoting research, these ambassadors could organize gatherings with like-minded individuals that would allow community members to interact with medical specialists in casual, culturally appropriate settings. In turn, this could cause participants to feel less intimidated while at the same time overcoming mistrust in physicians [18,31]. Importantly, involving the community in various phases of the research process can further enhance engagement and ensure that the conducted research aligns with the community’s needs and values [32]. In practice, research has shown that implementing community engagement principles can enhance research participation among ethnic minority patients by up to 80% [33].

### 3.4. Registering Ethnicity

Last but not least, enhancing research on ethnic differences is crucial for clarifying mechanisms underlying various cardiovascular diseases. However, self-reporting of ethnicity for research purposes has its limitations, as concerns about privacy or distrust in research may lead to lower participation rates among ethnic minorities, and, accordingly, to misrepresentation of the population [18]. Registering ethnicity by default at the start of care or research, assuming that it is necessary, while respecting privacy and cultural sensitivities, may contribute to the expansion of existing databases and make research more accessible [34]. A potential problem may be that scientists and healthcare professionals are reluctant to do so because they believe that ethnicity registration is not allowed by the EU General Data Protection Regulation and national privacy legislation. This is a widespread misunderstanding, as clarified through a comment in the British Medical Journal [35]. Educating healthcare professionals on the legal allowances and ethical considerations regarding ethnicity registration is essential to overcome this barrier.

Another hurdle on the way to better registration practices is that uniform classifications of ethnicity are lacking, which affects the reliability and consistency of research [10] and complicates the comparison of transnational study results. Establishing standardized definitions in future research is essential for enhancing the generalizability of findings. A sensible approach would be to define ethnicity and race based on standardized categories, in alignment with recommendations for the standardized collection of diversity data and taking into account updated guidelines on reporting diversity data [36,37].

Next to this, it is important to address potential implementation challenges such as institutional resistance and financial constraints. Governmental funding of transborder research projects like HELIUS [13] and RODAM [12], which concentrate on discovering ethnicity-based inequities across numerous diseases, could also aid in making research more valuable for the whole patient population.

## 4. Conclusions

In conclusion, a multifaceted improvement plan is needed to encourage ethnic minority involvement in clinical and observational research. Our proposed solutions, which align with findings in the existing literature, suggest that by addressing the unique barriers complicating the involvement of ethnic minorities in research can achieve a more representative and inclusive participant pool. This can ultimately enhance personalized healthcare for all individuals.

## Figures and Tables

**Figure A1:**
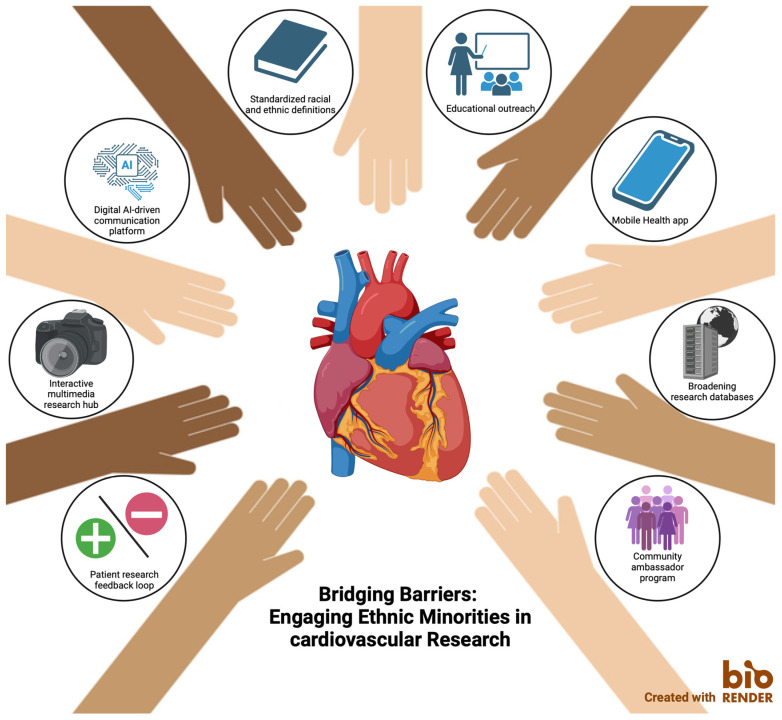
This graphical abstract illustrates solutions to address the underrepresentation of ethnic minorities in medical research. Our proposed solutions suggest that by addressing the unique barriers faced by ethnic minorities, clinical research can achieve a more representative and inclusive participant pool.

## Data Availability

No new data were created or analyzed in this study. Data sharing is not applicable.

## Appendix A

**Table A1:** Barriers and proposed solutions for ethnic minority underrepresentation in cardiovascular research.

Barrier	Proposed Solutions
Language barriers	Multilingual communication material
	AI-powered translation tools
	Simplified, linguistically appropriate communication
Healthcare mistrust	Trust-building approach
	Community ambassadors’ programs
	Community involvement in research development
Digital illiteracy	Interactive multimedia tools
	Interactive consent forms
	Mobile health applications
Lack of patient engagement	Feedback systems
	Gamification for engagement
Cultural sensitivities	Education on cultural beliefs and disease
	Dietitians offering culturally appropriate nutritional guidance
	Cultural competence education for healthcare professionals
Privacy and ethnicity registration concerns	Development of standardized reporting of ethnic background
	Educating health professionals on the General Data Protection Regulations regarding ethnicity data
	Default ethnicity registration
